# Specific adaptations are selected in opposite sun exposed Antarctic cryptoendolithic communities as revealed by untargeted metabolomics

**DOI:** 10.1371/journal.pone.0233805

**Published:** 2020-05-27

**Authors:** Claudia Coleine, Federica Gevi, Giuseppina Fanelli, Silvano Onofri, Anna Maria Timperio, Laura Selbmann

**Affiliations:** 1 Department of Ecological and Biological Sciences, University of Tuscia, Viterbo, Italy; 2 Department of Science and Technology for Agriculture, Forestry, Nature and Energy, University of Tuscia, Viterbo, Italy; 3 Italian National Antarctic Museum (MNA), Mycological Section, Genoa, Italy; Free University of Bozen-Bolzano, ITALY

## Abstract

Antarctic cryptoendolithic communities are self-supporting borderline ecosystems spreading across the extreme conditions of the Antarctic desert and represent the predominant life-form in the ice-free areas of McMurdo Dry Valleys, accounted as the closest terrestrial Martian analogue. Components of these communities are highly adapted extremophiles and extreme-tolerant microorganisms, among the most resistant known to date. Recently, studies investigated biodiversity and community composition in these ecosystems but the metabolic activity of the metacommunity has never been investigated. Using an untargeted metabolomics, we explored stress-response of communities spreading in two sites of the same location, subjected to increasing environmental pressure due to opposite sun exposure, accounted as main factor influencing the diversity and composition of these ecosystems. Overall, 331 altered metabolites (206 and 125 unique for north and south, respectively), distinguished the two differently exposed communities. We also selected 10 metabolites and performed two-stage Receiver Operating Characteristic (ROC) analysis to test them as potential biomarkers. We further focused on melanin and allantoin as protective substances; their concentration was highly different in the community in the shadow or in the sun. These results clearly indicate that opposite insolation selected organisms in the communities with different adaptation strategies in terms of key metabolites produced.

## Introduction

The Antarctic cryptoendolithic communities are microbial ecosystems that dominate the biology of most ice-free areas in Continental Antarctica. They were described for the first time in the McMurdo Dry Valleys, Southern Victoria Land [1], the largest ice-free area of the continent. The McMurdo Dry Valleys are a nearly pristine environment largely undisturbed and uncontaminated by humans and show remarkable peculiarities, representing an important analogue for the conditions of ancient Earth and Mars and a model environment for astrobiological studies [2–7]. These ice-free areas, dominated mostly by oligotrophic mineral soil and rocky outcrops [8, 9], due to the harshest conditions such as low temperatures (always below the freezing point) [10], strong and rapid thermal fluctuations, dryness, oligotrophy and high UV radiation, are dominated by cryptic microbial life-forms dwelling inside rocks [11]. For their unique geological and biological peculiarities, the McMurdo Dry Valleys, as a whole, are now designated as an ASMA (Antarctic Specially Managed Area), to assist planning and coordination of activities, improving cooperation between parties and minimise environmental impacts [12] and include five different ASPA (Antarctic Specially Protected Areas) to protect outstanding environments that require specific permits for entry. There, the prohibitive conditions are incompatible with active life on rock surfaces, and endolithic adaptation confers to microbes the chance to exploit a more protected niche inside rock porosity characterized by a milder and more stable microclimate [13–15]. Many different typologies of endolithic microbial communities have been described but the most complex and widespread in these areas are surely the cryptoendolithic lichen-dominated communities [1]. These are complex and self-supporting assemblages of phototrophic and heterotrophic microorganisms, including Bacteria, Chlorophyta and both free-living and lichen-forming fungi [1, 16] and, living at the edge of their physiological adaptability, represent the only chance for survival before extinction [11, 17].

The effect of global warming is dramatically intense at the Poles [18] and disturbance due to climate change may have irreversible effects on the weak equilibrium of these highly adapted microbial communities [19–21], but sensitive to any external perturbation [22]. Thus, improving our knowledge on functional capacity and multiple stress-responses of these ultimate ecosystems will give us important clues for monitoring and predicting any future variation due to climate change [19, 23]. Recent studies investigate the biodiversity of the McMurdo Dry Valleys [16, 24–27] and provided new insights on how the combination of environmental parameters of altitude and sea distance influence the distribution and the settlement of cryptoendolithic colonization [19–21, 28–29]. The key effect of the sun exposure in shaping the composition and abundance of functional groups of fungi in the Antarctic endolithic communities has been recently proved [19–21] as the northern exposed surfaces present considerably more favourable conditions than southern facing rocks [30–32] that, being in the shadow, may be too cold to allow biological activity.

Indeed, the capability to maintain biological activity depends on sufficient insolation on the rock surfaces allowing an efficient photosynthetic process; moreover, temperature became warmer for metabolic activity due the increasing of aw as consequence of melting of snow [33].

Antarctic cryptoendolithic microorganisms have to face several stresses simultaneously and they need to concurrently develop survival strategies to address external conditions. A conspicuous number of studies have been performed on the adaptation strategies on black fungi isolated from these communities, resulting highly resistant in terms of extremes of temperatures, acidity, osmotic stress and salinity, dehydration and irradiation [34–39]; besides, the stress-response mechanisms of whole metacommunity has not yet been investigated.

In this study, for the first time to our knowledge, metabolomics was successfully applied to Antarctic cryptoendolithic lichen-dominated communities to define adaptation strategies adopted by these communities to survive in these harshest conditions. We found different microbes’ response to sun exposure by modulating specific pathways; in particular, among the candidates to be considered biomarkers, the precursor metabolites of melanin and allantoin pathways were the most affected by sun exposure and we may consider these pathways to be directly involved to response to the environmental pressure.

## Materials and methods

### Samples collection

**Fig 1:**
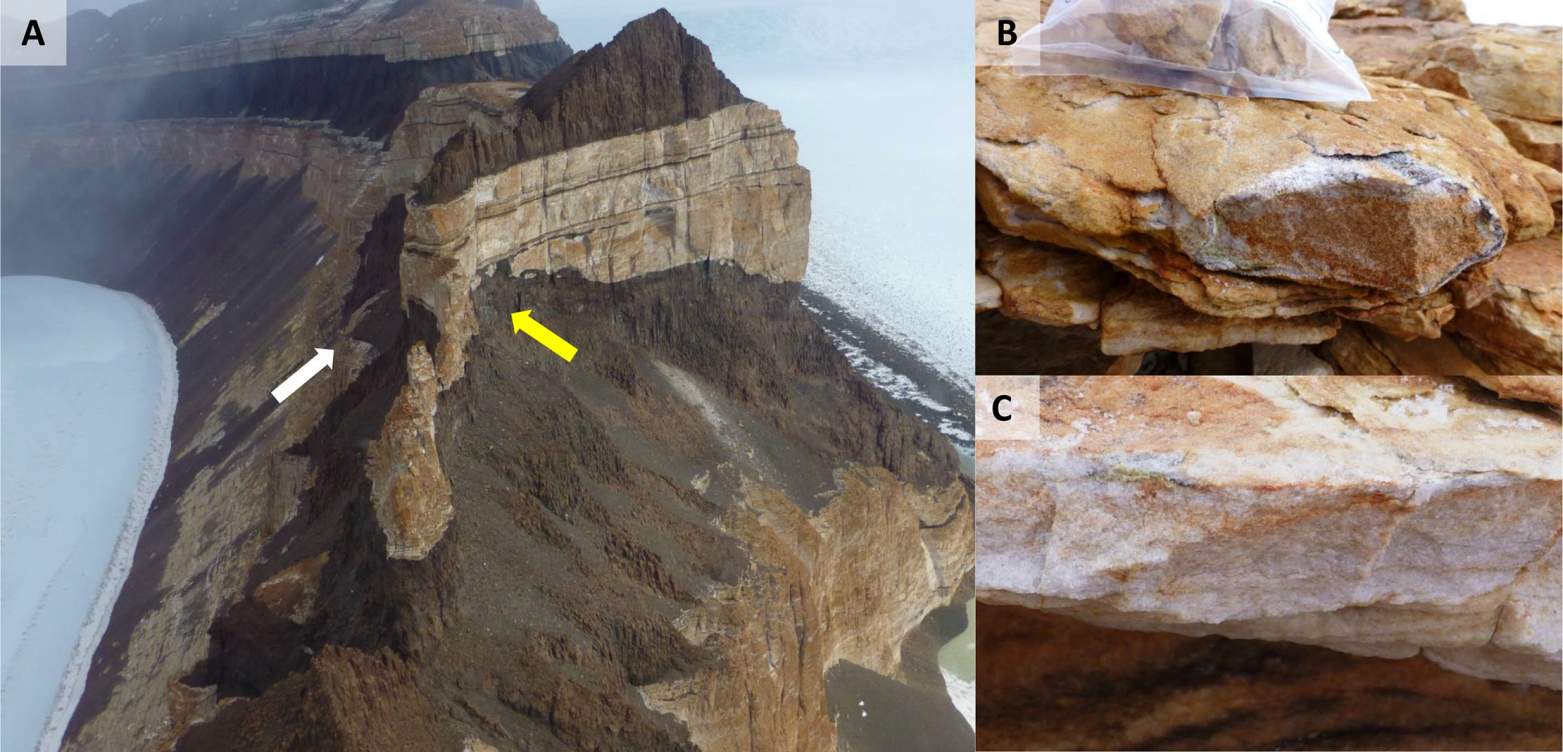
A) Finger Mt. landscape: the yellow arrow indicates the north exposed surface, while the white arrow indicates the south exposed surface; B) north exposed sandstone rock; C) south exposed sandstone rock.

Sandstones were collected in triplicate at Finger Mt. (1720 m a.s.l., McMurdo Dry Valleys, Southern Victoria Land, Continental Antarctica) both from north (77°45’0.93"S 160°44'45.2"E) and south (77°45’10"S 160°44'44.39.7"E) exposed surfaces by Laura Selbmann during the XXXI Italian Antarctic Expedition (Dec. 2015-Jan. 2016) (Fig 1). Samples have been collected in the frame of Italian National Antarctic Research Program (PNRA) projects. Sampling permits have been obtained for activity in Special Managed Areas (ASMA) and Special Protected Areas (ASPA) in compliance with the "Protocol on Environmental Protection to the Antarctic Treaty" Annex V, art.7.

The presence of endolithic colonization was assessed by direct observation *in situ* using magnification lenses. Rocks were excised using a geological hammer, placed in sterile bags and shipped at -20°C at the at University of Tuscia (Italy) where have been preserved at -20°C in the Mycological Section of the Italian Antarctic National Museum (MNA), until processing.

### Metabolites extraction

Classical metabolomics extraction protocol was adapted to rock sample. The frozen samples were powdered in liquid nitrogen. Ultrasonic crushing was performed at a low temperature, two times for 30 min. One gram of each rock was added to 3000 μl of a chloroform/methanol/water (1:3:1 ratio) solvent mixture stored at -20°C. Briefly, samples were vortexed for 5 min and left on ice for 2 h for total protein precipitation. Solutions were then centrifuged for 15 min at 15,000×g. and were dried to obtain visible pellets. Finally, the dried samples were re-suspended in 0.1 mL of RNA/DNAse free water, 5% formic acid and transferred to glass autosampler vials for LC/MS analysis. Extraction was performed in triplicate.

### Ultra-high-performance liquid chromatography

Twenty uL of each extracted supernatant sample were injected into an ultra-high-performance liquid chromatography (UHPLC) system (Ultimate 3000, Thermo): samples were loaded onto a Reprosil C18 column (2.0mm× 150 mm, 2.5 μm-DrMaisch, Germany) for metabolite separation. Chromatographic separations were made at flow rate of 0.2 ml/min. A 0–100% linear gradient of solvent A (ddH2O, 0.1% formic acid) to B (acetonitrile, 0.1% formic acid) was employed over 20 min, returning to 100% A in 2 min and holding solvent A for a 6-min post time hold. The UHPLC system was coupled online with a Q Exactive mass spectrometer (Thermo Fisher, Rockford, IL) scanning in full MS mode (2 μ scans) at resolution of 70,000 in the 67 to 1,000 m/z range, with 3.8 kV spray voltage, 40 sheath gas, and 25 auxiliary gas. The system was operated in positive ion mode. Calibration was performed before each analysis against positive or negative ion mode calibration mixes (Thermo Fisher) to ensure error of the intact mass within the sub ppm range. Metabolite assignments were performed using MAVEN v5.2 [40]. Each replicate was analysed separately and a p-value < 0.01 was used to infer significance for all abundance comparisons between sets of triplicates.

### Data elaboration and statistical analysis

Replicate files were processed through MAVEN v5.2, enabling rapid and reliable metabolites quantitation from multiple reaction-monitoring data or high-resolution full-scan mass spectrometry data. Mass Spectrometry chromatograms were created for peak alignment, matching and comparison of parent and fragment ions with tentative metabolite identification within a 2-p.p.m. mass-deviation range between the observed and expected results against an imported KEGG database [41]. To visualize the number of significant changes m/z values between metabolites datasets, a Volcano plot were created using the MetaboAnalyst 4.0 software (http://metpa.metabolomics.ca/). Raw data were normalized by sum and auto-scaling in order to increase the importance of low-abundance ions without significant amplification of noise. This type of plot displays the fold change differences and the statistical significance for each variable. The log of the fold change is plotted on the x-axis so that changes in both directions (up and down) appear equidistant from the centre. The y-axis displays the negative log of the p-value from a two-sample t-test. False discovery rate (FDR) [42] were used for controlling multiple testing.

Metabolites subject to major changes were displayed and Receiver Operator Characteristic (ROC) curves for each of them was calculated using the same software to evaluate potential metabolites to be considered as biomarkers. A ROC diagram plots the true positive rate (sensitivity) of a test on the y-axis against the false positive rate (100-specificity) on the x-axis, yielding the ROC area under the curve (AUC). Within this model, the ROC curves used 5-fold cross validation. AUC can be interpreted as the probability that a test or a classifier will rank a randomly chosen positive instance higher than a randomly chosen negative one. If all positive samples are ranked before negative ones (i.e. a perfect classifier), the AUC is 1.0. An AUC of 0.5 is equivalent to randomly classifying subjects as either positive or negative (i.e. the classifier is of no practical utility).

Metabolic pathways were displayed with Graphpad Prism v5.01 (Graphpad Software Inc) and statistical analyses were performed with the same software (p < 0.05). Data are presented as mean ± SD. Differences were considered statistically significant at *p < 0.05 and further stratified to **p < 0.01, respectively.

## Results

### Shaping in metabolites composition

An untargeted metabolomics analysis was performed on six differently exposed rocks and more than 10,000 peaks per sample were referred to the KEGG database; among them, 2,807 metabolites were analysed more precisely and identified.

**Fig 2:**
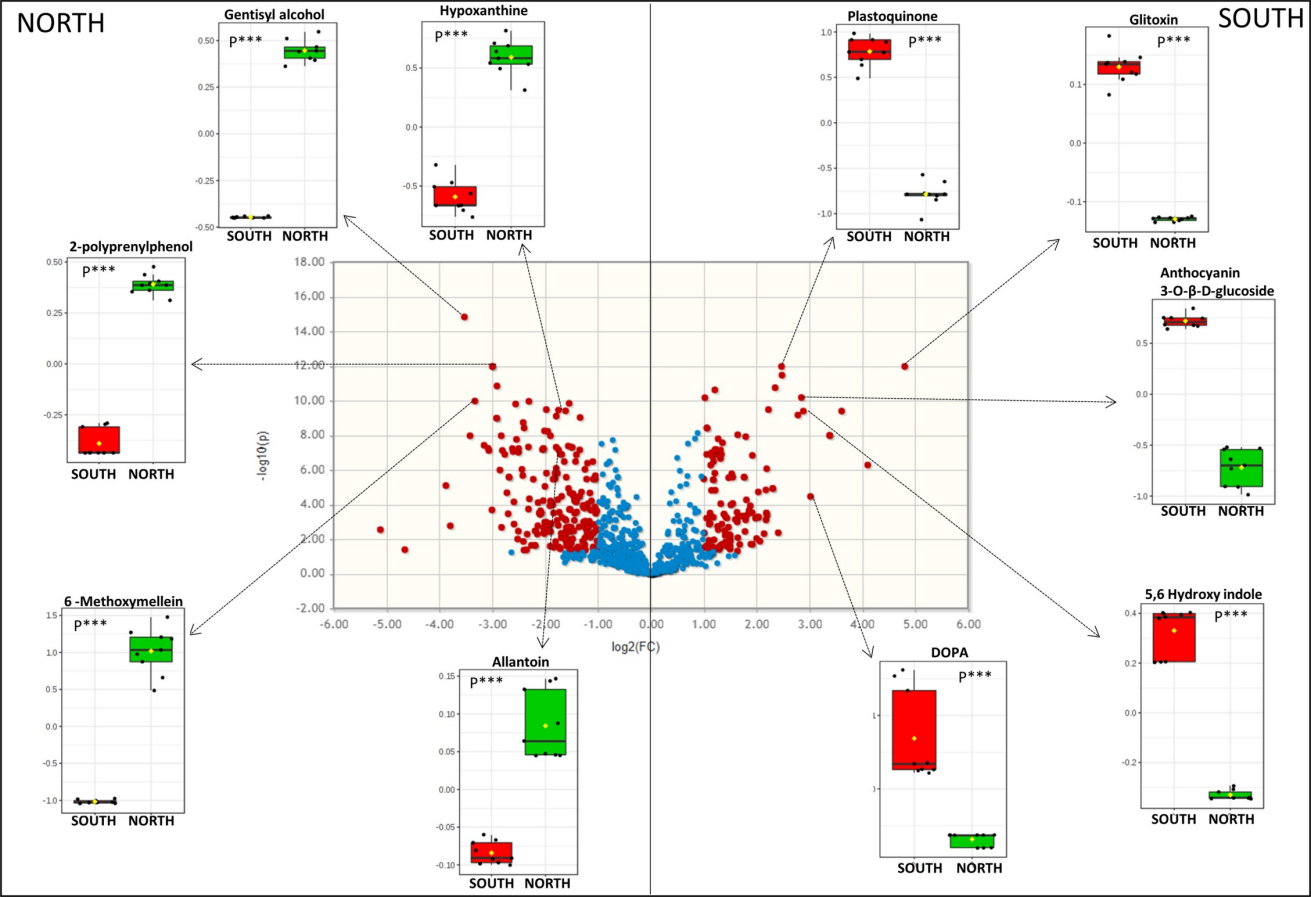
Volcano plot analysis of metabolic changes Finger Mt. north and Finger Mt. south. Each point on the volcano plot was based on both p-value and fold-change values, and in this study these two values were set at 0.05 and 2.0, respectively. The points which satisfy the condition p < 0.05 and fold change > 2.0 appear in red and are marker candidates, whereas the others appear in blue that are not significant. The potential biomarkers between experimental groups were annotated with their matched metabolite names. Bar plots show the original values (mean +/- SD). Differences were considered statistically significant at *p < 0.05 further stratified to **p < 0.01 and ***p < 0.001 respectively.

The significant discriminating metabolites were identified using the Volcano plot analysis (Fig 2); the univariate analysis identified significant accumulation of specific metabolites; most of them were expressed in rocks collected in south exposure, while few others were overexpressed in north exposed rocks.

Based on the specific criteria, 206 metabolites were significantly upregulated in Antarctic cryptoendolithic communities of rocks exposed to the north whereas 125 were significantly upregulated in the south exposed ones (Fig 2). S1 Table summarized all metabolites identified considering the following parameters: p-value, FC (fold change), log2 (FC fold change), and -log10 (p-value).

**Fig 3:**
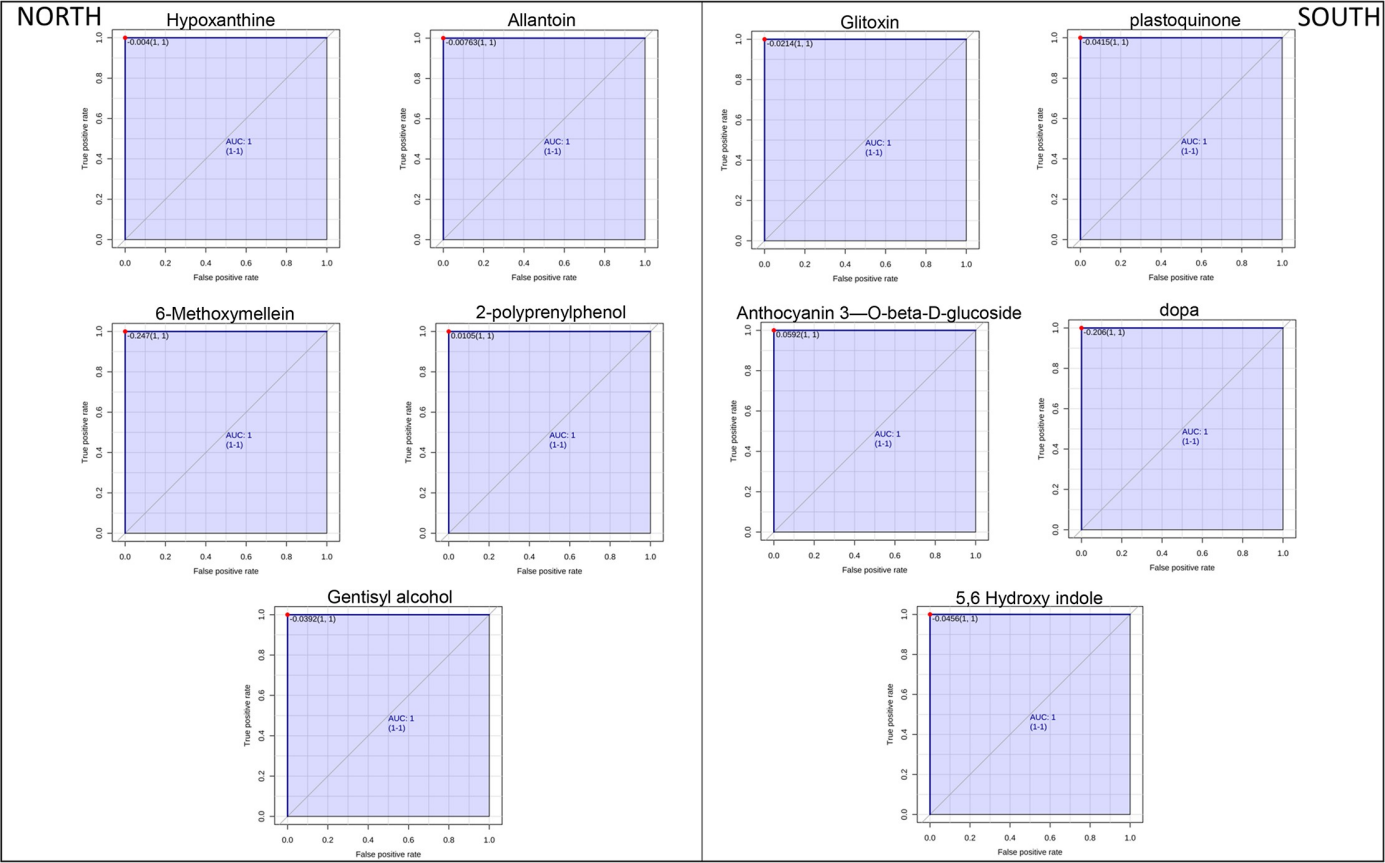
Area under the receiver-operating characteristic curves (AUROC) of the significant metabolite (with AUROC value > 0.9) identified 5 metabolites (Allantoin, Hypoxanthine, 6-Methoxymellein, 2-Polyprenylphenol and Lomefloxacin hydrochloride) as significantly elevated in Finger Mt. north samples and 5 metabolites (Gliotoxin, Plastoquinone, Anthocyanin 3-O-β-D-glucoside, 5–6 Dihydroxyindole and L-DOPA) significantly elevated in Finger Mt. south samples. They are candidate biomarkers with excellent value (AUC = 1; CI = 1−1).

Candidate markers were selected, using a Receiver Operating Characteristic (ROC) curve analysis, by examining the volcano plot and considering a fold change threshold of 2 and p-value < 0.05. Five significantly upregulated metabolites in the north-exposed communities (*Allantoin*, *Hypoxanthine*, *6-Methoxymellein*, *2-Polyprenylphenol* and *Gentisyl alcohol*) and five metabolites increased significantly in those facing south (Fig 2) (*Gliotoxin*, *Plastoquinone*, *Anthocyanin 3-O-β-D-glucoside*, *5–6 Dihydroxyindole* and *L-DOPA*) showed an AUC = 1 that represented a perfect ROC curve according to the accepted classification of biomarkers [43] (Fig 3).

Based on ROC analysis, changes in Allantoin (northern rock surface) and L-DOPA (southern rock surface) expression were further analysed to clarify their roles in the Antarctic cryptoendolithic communities.

### Allantoin pathway

**Fig 4:**
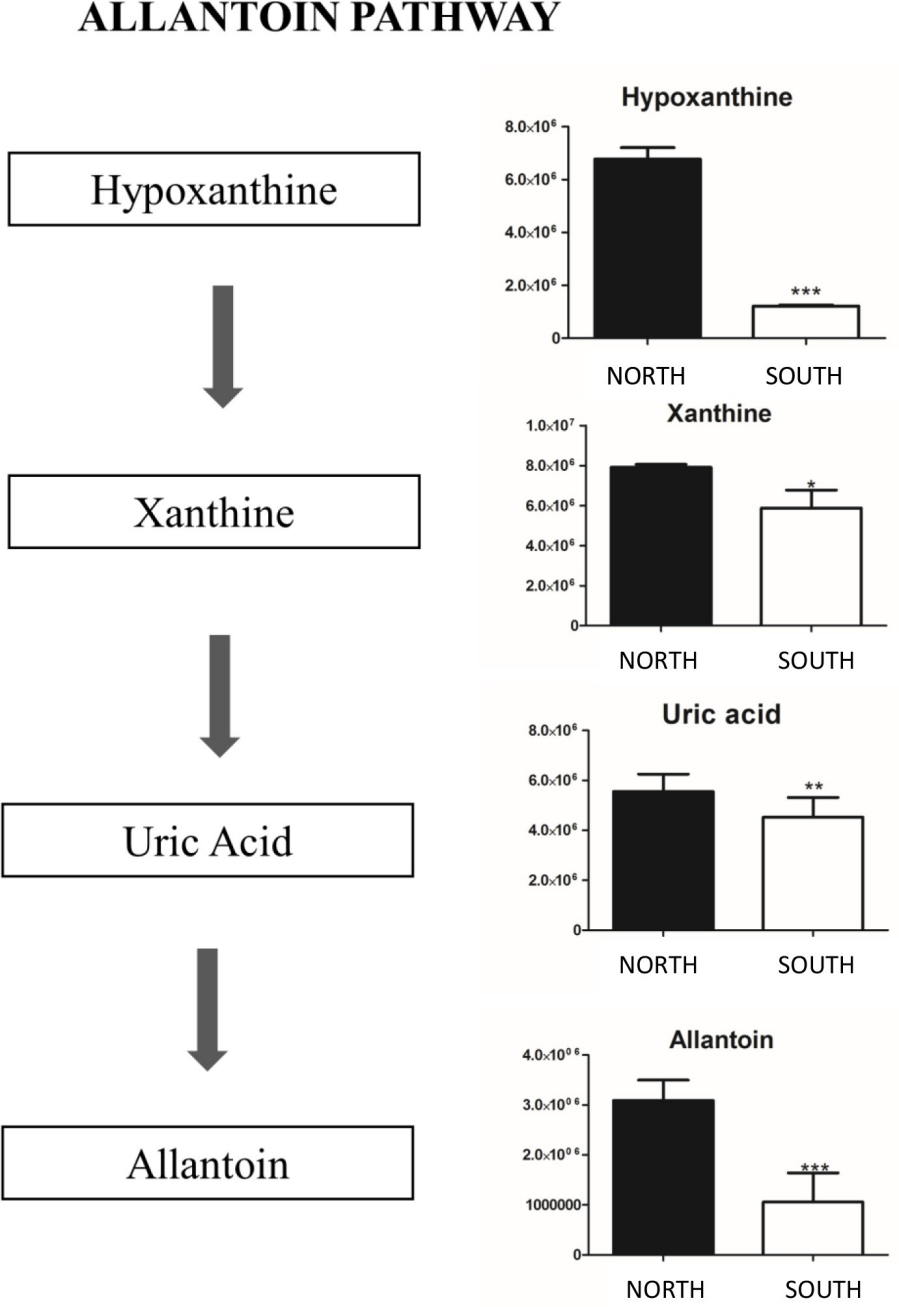
Allantoin pathway. Total amount of allantoin pathway intermediates appears to be increased in Finger Mt. north. Metabolites are expressed as the mean ± SD concentration over Finger Mt. north *p < 0.05, **p < 0.01 against Finger Mt. south.

Fig 4 showed the significant changes observed in the intermediates allantoin biosynthesis.

Biosynthesis starts from purine metabolism degradation (Hypoxanthine and Xanthine) and the intermediate Uric Acid. All allantoin pathway intermediates were stable and more abundant in the north exposure; while, an intermediates fluctuation was observed in the south samples. The amount of uric acid and allantoin decrease significantly in south samples (p < 0.05).

### Melanin pathway

**Fig 5:**
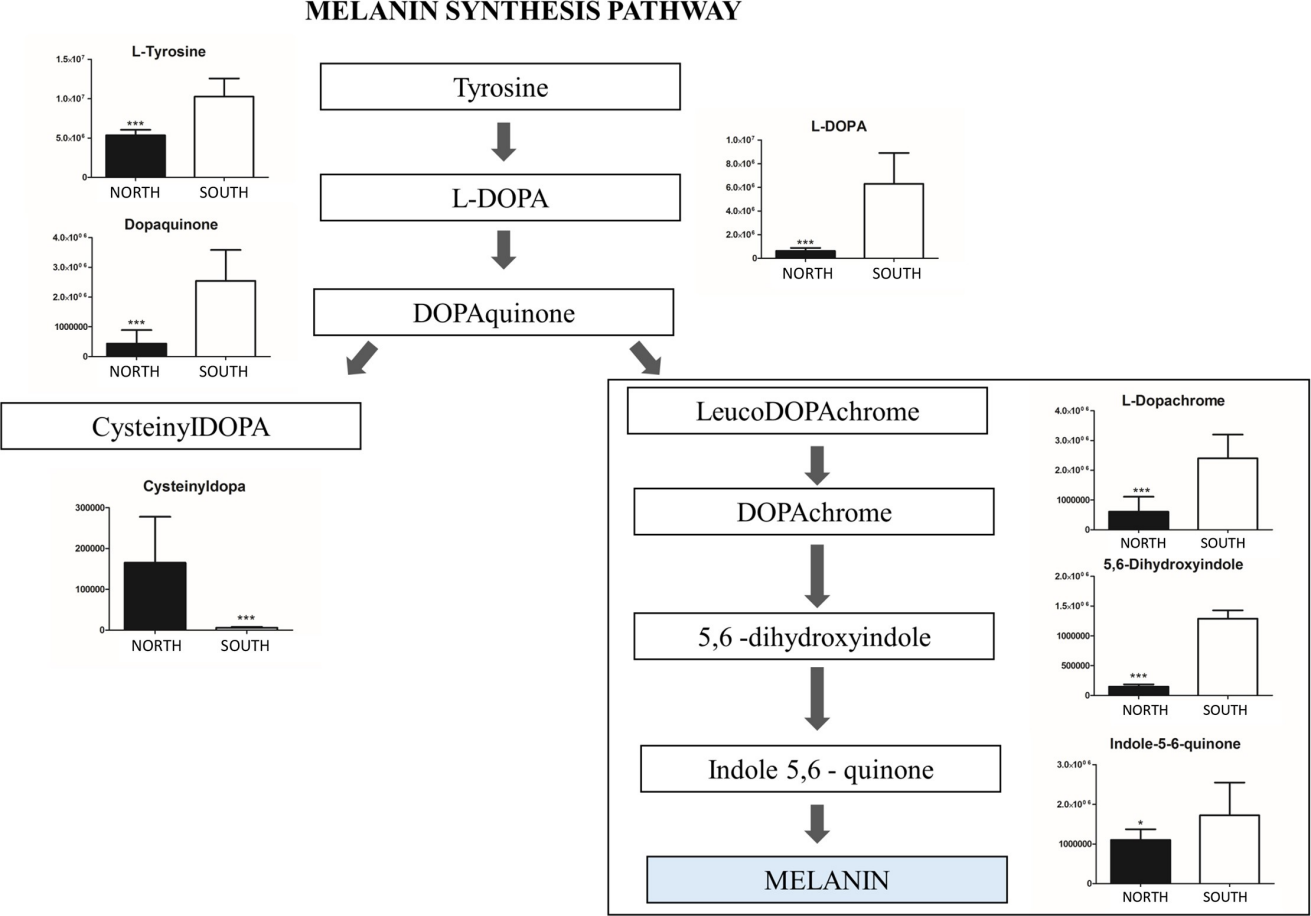
Melanin synthesis pathway. The conversion of tyrosine to melanin involves several steps and we found overall up-regulation of Finger Mt. south intermediates whereas Cysteinyl DOPA decreases, confirming that samples from south increase the synthesis of melanin. Metabolites are expressed as the mean ± SD concentration over Finger Mt. north *p < 0.05, **p < 0.01 against Finger Mt. south.

In Fig 5 the melanin biosynthetic pathway is reported. Melanin is produced starting from oxidation of amino acid L-Tyrosine. With our mass spectrometry techniques, it is possible to identify the tyrosine metabolism intermediates. The rate-limiting initial step in the biosynthesis of melanin is the hydroxylation of tyrosine to L-3,4-dihydroxyphenylalanine (DOPA) and its immediate subsequent oxidation to DOPAquinone (DQ). Dopaquinone went through instantaneous intramolecular nonenzymatic cyclization forming leucochrome, which is rapidly oxidized by dopaquinone to dopachrome at the level of DOPAquinone point of switch. The pathway lead to the synthesis of melanin instead of cysteinyl DOPA. Since all the intermediates (leucoDOPAchrome, DOPAchrome, 5–6 dihydroxy indole, Indole 5,6 quinone) reached the maximum level in south exposed samples (p < 0.05), we have considered the melanin biosynthesis significantly enhanced in southern exposed rocks.

## Discussion

Understanding the mechanisms driving functional and ecological processes in the extremes remains a major challenge [44–46], particularly since environmental stressors are often associated with diminished ecosystem capacity and functionality [47–50]. This is particularly true in hot and cold desert ecosystems such as the Antarctic Dry Valleys (Continental Antarctica) are accounted as the coldest and most hyper-arid desert on Earth and the closest Mars analogue on Earth [5, 6].

Metabolites are the result of both biological and environmental factors, and provide great potential to bridge knowledge of genotype and phenotype. In contrast to targeted metabolomics, non-targeted approaches offer the potential to determine biomarkers. The idea that untargeted mass spectrometry (MS)-based metabolomics analysis will result in a large list of ‘identified’ small molecules that can be mapped to networks and pathways is often assumed [51].

In this study, we considered rocks collected at opposite exposure, accounted among the principal driving factor for settlement and shaping stress-response in cryptoendolithic life-style; indeed, recently, sun exposure was found to be critical in shifting community composition, taxon abundance, and distribution of functional groups of fungi in Antarctic endolithic communities [21, 28], shaping these ecosystems more than other abiotic parameters previously considered (e.g. altitude). Besides, the differences in the ability to respond to stress between northern and southern exposed communities, if any, have never been investigated.

In our study, the concentration and composition of metabolites presented a clear pattern of correlation across different exposed surfaces of the same mountain, where the changes in the microbial community metabolic profiles following sunlight deprivation were evident. Overall, the remarkable variability observed across exposures indicated that this environmental parameter can be considered as driving factor shaping the stress-response strategies in the Antarctic cryptoendolithic communities. Indeed, through the Volcano Plots, we highlighted the differential abundance of metabolites influenced by this parameter. In total, we identified 206 metabolites that were significantly expressed in north exposed rocks and 125 in southern samples, as shown in Fig 2 (p < 0.01, S1 Table), where conditions are more severe. The ability to thrive at temperature and conditions, such as those associated to southern-exposed rocks, requires a vast array of adaptations to maintain the metabolic rates to guarantee active life; thus, microbial communities may have evolved diverse and tougher mechanisms to successfully exploit southern surface where conditions are harsher compared with north exposed faces [52, 53], by up or down-regulating the expression of a significant number of genes encoding cold-shock proteins (CSPs) process namely cold-shock response. Genes that are strongly up-regulated in response to cold exposure include a number of cold-induced RNA helicases, molecular chaperones, heat shock proteins and genes associated with sugar transport and metabolism [54, 55] and genes encoding antioxidative enzymes such as catalases and superoxide dismutase [56].

Recent studies reported an up-regulations of a highly diverse set of metabolic features to cope cold-associated stresses; for instance, in an Antarctic alga *Chlamydomonas* sp., RNA helicase genes were up-regulated after low temperature exposure [57, 58], while, photosynthetic metabolism in *Chaetoceros neogracile*, isolated from Antarctic ocean, was found highest at 4°C than at room temperature [59]; earlier, a protease was found to be produced in higher amount at low temperature in the Antarctic yeast *Candida humicola* [60]. However, given the many different adaptive mechanisms that are used by different psychrophiles, many other strategies for survival at cold temperatures are still to be discovered [61].

Additionally, the volcano plot analysis, coupled with Receiver Operating Characteristics (ROC) curves analysis, was employed to explore the chemical markers that contributed most to the difference in profiles across sampled area, determining which compounds were of interest. Among the detected metabolites, we produced a hit of metabolites specimens and identified at least 10 metabolites that were specifically correlated to a specific condition. The ROC curves (Fig 3), determined that *allantoin*, *hypoxanthine*, *6-methoxymellein*, *2-polyprenylphenol*, and *Gentisyl alcool* were the most sensitive and specific biomarkers for north exposed rocks (AUC = 1), while *l-dopa*, *5-6-dihydroxindole*, *gliotoxin*, *plastoquinone*, and *anthocyanin* were unique for south exposed microbial communities (AUC = 1). All these metabolites are intermediates of crucial pathways responsible for stress-adaptation: among the north-related biomarkers, *6-methoxymellein* has been reported to be involved in UV-radiation response in plant systems [62]; *2-polyprenylphenol* (member of the class of phenols and precursor of ubiquinone) plays an important role in plant resistance and in plant growth and development, through the biosynthesis compounds that act as antioxidants [63]. Their synthesis is generally triggered in response to biotic/abiotic stresses and especially under salt stress conditions; they take part in defence responses during infection, excessive sun exposure, injuries and heavy metal stress [64].

Instead, among biomarkers responsible for stress-response in south-exposed communities, *gliotoxin* is involved in protecting *Aspergillus fumigatus* against oxidative stress against the reactive oxygen species (ROS) [65, 66]; indeed, *gliotoxin* is an epipolythiodioxopiperazine (ETP), a toxic secondary metabolites made only by fungi with molecular mass 326 Da and contains a disulphide bridge which can undergo repeating cleavage and reformation, thereby resulting in a potent intracellular redox activity [67]. *Plastoquinone* participates in the metabolism of various important chemical compounds, acting as antioxidants, involved in plant response to salinity stress [68, 69]. *Anthocyanins* are well-known water‐soluble pigments found in all plant tissues throughout the plant kingdom, involved in UV-B radiation, water stress and particularly cold temperatures [70], as reported in *Arabidopsis* [71, 72]. Indeed, McKown et al. [73] suggested a sort of commonality between anthocyanin biosynthesis and freezing tolerance as *Arabidopsis* mutants deficient in freezing tolerance were unable to accumulate anthocyanins, resulting in an increased tolerance of cool temperatures.

We further focused on *allantoin* and *hypoxanthine* over expressed in north, and *DOPA* and *5–6 hydroyndole* over expressed in south since they can play a role in different stress responses in the endolithic communities of the two-opposite exposed surfaces. The *allantoin* plays an important protective role to excessive sun exposure; this pathway resulted over- expressed in the north exposed communities, where climatic conditions are more buffered and allow a successful settlement of endolithic colonization. Besides, the degree of insulation is much higher than in the south and photosynthetic organisms such as cyanobacteria and algae need to be protected by UV irradiation. *Allantoin* is also produced in plants, providing protective function by blocking UV-B before it reaches cells, the consequences of UV-B exposure such as repairing damaged DNA [74]. Metabolomics studies revealed allantoin as a major purine metabolite in *Arabidopsis thaliana*, *Oryza sativa* and other species under various stress conditions such as drought [75–77], high salinity [78, 79], low temperature [80] and nutrient constraints [81–83], environmental characteristics that are exacerbated in Antarctic ice-free areas.

Conversely, the south exposed surface is in more prohibitive climatic conditions and in the shadow over the entire year, with limited possibility for metabolic activity and the need of a super-protective shield is of utmost importance for the survival of the community as a whole. In this environment, *melanin* pathway resulted more expressed that in the northern surface. *Melanins* are ancient biological pigments found in all kingdoms of life and, in particular in the fungal kingdom, are present across all phyla with remarkable physicochemical properties, allowing them to perform diverse functions in biological systems [84–87]. Mainly, two pathways of melanin synthesis are found in fungi: most of them synthesize melanin via the DHN (1,8-dihydroxynaphthalene) pathway, whereas few species are able to produce melanin via l-DOPA (3,4-dihydroxyphenylalanine) in a pathway that resembles mammalian melanin biosynthesis [88]. Recently, it was found evidence for the production of a water-soluble pigment similar to pyomelanin by *Penicillium chrysogenum* [89]. Despite the wide number of melanotic species and biosynthetic pathways for melanin production, the exact *melanins* produced by the Antarctic cryptoendolithic fungi are still unknown; besides, it has been suggested that all fungal *melanins*, regardless of their precursor, may share similar physicochemical properties [90]. Some fungal species, constitutively melanized are referred to as black, dematiaceous, micro colonial or meristematic fungi [34] with a worldwide distribution, typically colonizing harsh environmental niches not suitable to most life forms such as saltpans, hydrocarbon-contaminated sites, exposed bare rocks and monuments, icy habitats, deserts, solar panels and building roofs [35, 91–96]. This group of fungi is also invariably present in Antarctic cryptoendolithic communities [20, 97–100] where they form a black barrier just above the photobiont’s stratification, supplying protection to algae by dissipating excessive sunlight [101].

These results are confirmed in a recent study where the authors showed that black fungi were invariably predominant in communities sampled in southern exposed rocks [21]. Actually, the highly melanized cell wall in black fungi plays a crucial protective role also to other stresses in addition to sunlight and UV radiation such as low temperature, high osmotic pressure, oxidative stresses, low water activity and nutrient availability [102–107] that can be more intense in south exposed surfaces.

## Conclusions

This first contribution allowed us starting to discern the functionality of these unique microbial communities, demonstrating how the metabolic response shifts across variations due to sun exposure, and giving insights on the capabilities of these ultimate ecosystems to sustain their growth and survival in such harsh environments. We validated application of an untargeted metabolomics method that provides optimal identification of a wide range of metabolites to detect metabolic changes in the main pathways, determining which products are being released into the environment. We conclude that further investigations on changes to the metabolic profiles may have potential for use as early indicators, to forecast the impact of Global Change that is challenging for the structure and functioning of cryptoendolithic ecosystems in Antarctic deserts.

## Supporting information

S1 Table The features displayed in the table are ranked based on area under Receiver Operating Characteristic (ROC) curve (AUROC), T-statistics (p value < 0.05) and Log2 fold change (FC). (PDF)
