# Supplementary material for: Specific adaptations are selected in opposite sun exposed Antarctic cryptoendolithic communities as revealed by untargeted metabolomics
Source: PLoS One. 2020 May 27;15(5):e0233805. doi: 10.1371/journal.pone.0233805 (PMC7253227; doi:10.1371/journal.pone.0233805)
Supplement: S1 Table — (PDF) [file pone.0233805.s001.pdf]

NORTH SUN EXPOSED ROCK SAMPLE				
**Metabolite**	**FC**	**log2(FC)**	**p value**	**-log10(p)**
Allantoin	5.9232	-2.56	0.001133	2.95
Hypoxanthine	0.0657	-3.92	0.011882	1.24
Falcarinol	0.0028	-8.48	6.92E-05	4.16
Aricine	0.0064	-7.29	6.92E-05	4.16
5-Hydroxy-L-tryptophan	0.0841	-3.57	6.92E-05	4.16
Nicotyrine	0.1204	-3.05	0.000108	3.97
1-Hydroxy-2-aminoethylphosphonate	0.3102	-1.69	0.000333	3.48
Scutellarein	0.0033	-8.24	0.000334	3.48
Thiethylperazine	0.0037	-8.10	0.000334	3.48
Miraxanthin-III	0.0493	-4.34	0.000334	3.48
2-Oxo-7-methylthioheptanoic acid	0.4287	-1.22	0.000334	3.48
4-4-Difluoro-17beta-hydroxyandrost-5-en-3-one propionate	0.1054	-3.25	0.000449	3.35
Tauropine	0.4865	-1.04	0.000523	3.28
6-Methoxymellein	0.198	-2.34	0.000556	3.26
Istamycin B1	0.0063	-7.31	0.000647	3.19
Broussonin C	0.0021	-8.92	0.000854	3.07
Methyl	0.0641	-3.96	0.000993	3.00
Cyclohexylsulfamate	0.0936	-3.42	0.001033	2.99
Simazine	0.0503	-4.31	0.001074	2.97
alpha-Zearalanol	0.315	-1.67	0.001246	2.90
Sarmentosin	0.1249	-3.00	0.001626	2.79
20-21-21-Trifluoro-3-methoxy-19-nor-17alpha-pregna	0.2104	-2.25	0.001626	2.79
Tween 80	0.0001	-13.15	0.001691	2.77
Rutecarpine	0.3899	-1.36	0.001757	2.76
Butoctamide hydrogen succinate	0.1069	-3.23	0.002254	2.65
Lomefloxacin hydrochloride	0.3548	-1.50	0.002516	2.60
2-Polyprenylphenol	0.001	-9.92	0.00252	2.60
RU 5135	0.0008	-10.25	0.002613	2.58
Curacin A	0.1531	-2.71	0.002625	2.58
Penicillin K	0.0013	-9.56	0.002754	2.56
Tolbutamide	0.1754	-2.51	0.00302	2.52
Bethanechol	0.0052	-7.59	0.003458	2.46
N-Acetyl-ala-ala-ala-methylester	0.0333	-4.91	0.00371	2.43
4-4-Disubstituted cyclohexenone	0.3806	-1.39	0.003716	2.43
2-Acetamidofluorene	0.0737	-3.76	0.003788	2.42
Sertraline	0.0299	-5.06	0.00386	2.41
DL-Pipecolic acid	0.2099	-2.25	0.003993	2.40
Candimine	0.1098	-3.19	0.004085	2.39
Sterol 3-beta-D-glucoside	0.1324	-2.92	0.004374	2.36
2-Demethylmenaquinone	0.0080	-6.97	0.004435	2.35
thymine	0.3775	-1.41	0.004541	2.34
Carisoprodol	0.4169	-1.26	0.004541	2.34
Sarothralin	0.0042	-7.91	0.005135	2.29
Harzianopyridone	0.0007	-10.56	0.005297	2.28
Nalpha-Benzyloxycarbonyl-L-leucine	0.0716	-3.80	0.005493	2.26
Questinol	0.0007	-10.45	0.005974	2.22
4-4--DiphenylethenylidenebisN-N-dimethylbenzenamine	0.0095	-6.72	0.006104	2.21

Dibenza-hanthracene	0.1382	-2.86	0.006125	2.21
D-Galactosyl-1-3-beta-D-galactosyl-1-4-beta-D-glucosyl-R	0.3787	-1.40	0.006218	2.21
Tolylacetonitrile	0.1533	-2.71	0.006253	2.20
Atrazine	0.0555	-4.17	0.006405	2.19
Thiamine aldehyde	0.0034	-8.19	0.006838	2.17
2-Hydroxyiminostilbene	0.0625	-4.00	0.007567	2.12
UH-301	0.4011	-1.32	0.008269	2.08
Indinavir sulfate	0.0980	-3.35	0.008747	2.06
4-2-Pyrazinylethenylphenol	0.0721	-3.79	0.00881	2.06
3-6-8-Trimethylallantoin	0.3982	-1.33	0.009825	2.01
UMP	0.0013	-9.62	0.009938	2.00
**SOUTH SUN EXPOSED ROCK SAMPLE**				
L-DOPA	22.704	4.50	0.0278	1.56
Dihydroxyindole	38.948	5.29	0.00269	2.58
Glitoxin	293.87	8.20	0.0000	6.96
p-Glucosyloxymandelonitrile	651.36	9.35	0.0000	6.96
1-6-Dinitropyrene	257.84	8.01	0.0000	4.89
Scandoside methyl ester	506.65	8.98	0.0000	4.89
citrulline	2.9662	1.57	0.0001	4.16
Dihydromethysticin	3.6798	1.88	0.0001	4.16
3-Phenylpropyl acetate	4.1601	2.06	0.0001	4.16
2-2-Dimethyl-3-4-bis4-methoxyphenyl-2H-1-benzopyran-7-ol	30.301	4.92	0.0001	4.10
N4-Phosphoagmatine	3.4328	1.78	0.0001	4.01
Phytosphingosine	4.3557	2.12	0.0001	4.01
hydroxyphenylpyruvate	15.333	3.94	0.0001	3.88
1-Hydroxyalkyl-sn-glycerol	5.4921	2.46	0.0001	3.87
Pipobroman	21.518	4.43	0.0001	3.83
11-Deoxocucurbitacin I	41.935	5.39	0.0002	3.77
Sulfadoxine	4.9139	2.30	0.0002	3.71
Chrysanthetriol	2.7453	1.46	0.0002	3.61
Warburganal	4.052	2.02	0.0002	3.61
6-Imino-5-oxocyclohexa-1-3-dienecarboxylate	1841.3	10.85	0.0003	3.59
Streptobiosamine	6.8079	2.77	0.0003	3.55
2-Aminomuconate semialdehyde	14.296	3.84	0.0003	3.55
Homostachydrine	84.149	6.39	0.0003	3.51
Huperzine B	2.6191	1.39	0.0003	3.50
Loperamide	5.7924	2.53	0.0003	3.50
Piperidine	14.745	3.88	0.0003	3.50
N-Acetylneuraminate	20.403	4.35	0.0003	3.50
Stachyose	29.427	4.88	0.0003	3.50
Gla protein precursor	2.7942	1.48	0.0003	3.48
N-Cyclopropylammelide	3.5946	1.85	0.0003	3.48
Sarcostin	3.6927	1.88	0.0003	3.48
Plastoquinone	4.6089	2.20	0.0003	3.48
Coleonol	5.2495	2.39	0.0003	3.48
Phosphinothricin	8.9336	3.16	0.0003	3.48
Vicine	14.51	3.86	0.0003	3.48
Arbutin	14.519	3.86	0.0003	3.48
L-Serine-phosphoethanolamine	24.923	4.64	0.0003	3.48
Oxidized Renilla luciferin	74.086	6.21	0.0003	3.48

Bowdichione	2618.1	11.35	0.0004	3.44
Dehydrofalcarinol	4.0094	2.00	0.0004	3.43
Cordycepin	25.462	4.67	0.0004	3.37
PD 123319	2.8655	1.52	0.0005	3.33
Lividomycin B	11.457	3.52	0.0006	3.26
6beta-17beta-Dihydroxyandrost-4-en-3-one diacetate	3.0057	1.59	0.0006	3.26
Buclizine	3.3833	1.76	0.0006	3.26
9-Fluoro-11beta-hydroxy-16beta-methylandrosta-1-4-diene-3-	3.8093	1.93	0.0006	3.26
Benzenamine sulfate	3.917	1.97	0.0006	3.26
D-Gal alpha 1-6D-Gal alpha 1-6D-Glucose	15.453	3.95	0.0006	3.26
Anthocyanin 3--O-beta-D-glucoside	21.16	4.40	0.0006	3.26
Cycloheximide	2.3778	1.25	0.0006	3.25
N3--Acetyl-2-deoxystreptamine antibiotic	14.392	3.85	0.0006	3.25
CDP-choline	1975.2	10.95	0.0006	3.19
Adifoline	3.6603	1.87	0.0007	3.14
Neurosporaxanthin	2084.1	11.03	0.0007	3.13
7-Methyladenine	16.63	4.06	0.0007	3.13
4-Amino-2-hydroxylamino-6-nitrotoluene	591.8	9.21	0.0008	3.10
Melibiitol	2.197	1.14	0.0008	3.08
Glycosyl-4-4--diaponeurosporenoate	2.6635	1.41	0.0008	3.07
Formylanthranilate	13.191	3.72	0.0008	3.07
Guanidoacetic acid	2.7054	1.44	0.0010	3.01
Cytochrome c S-methylmethionine	2.1493	1.10	0.0010	3.00
Nocardicin G	12.139	3.60	0.0010	3.00
Benzoapyrene-7-8-oxide	25.455	4.67	0.0010	3.00
Diazinon	15.334	3.94	0.0010	2.98
1-Alkyl-2-acylglycerophosphoethanolamine	9.1331	3.19	0.0011	2.94
Magnocurarine	38.864	5.28	0.0012	2.94
Evadol hydrochloride	6.1497	2.62	0.0012	2.93
Chloroprocaine	2.5138	1.33	0.0012	2.93
Primin	3.5421	1.82	0.0012	2.93
7-Acetyloxy-3-3-pyridinyl-2H-1-benzopyran-2-one	11.732	3.55	0.0012	2.93
N-Isopropylammelide	10.028	3.33	0.0012	2.92
5-Ureido-4-imidazole carboxylate	3.9508	1.98	0.0012	2.91
Flunisolide	2.6014	1.38	0.0012	2.90
Compound VS	3.5434	1.83	0.0013	2.90
Amodiaquine	3.9762	1.99	0.0013	2.87
Indolebutyric acid	6.3474	2.67	0.0014	2.87
Cinnamaldehyde	2.6398	1.40	0.0014	2.86
guanine	6.4786	2.70	0.0014	2.85
Estradiol 17beta-cyclopentylpropionate	3.2215	1.69	0.0014	2.85
Lasiocarpine	3.4909	1.80	0.0014	2.84
Tetrahydrozoline	132.78	7.05	0.0015	2.83
1-3-4-Dihydroxyphenyl-5-hydroxy-3-decanone	3.1305	1.65	0.0016	2.81
xanthine	2.5364	1.34	0.0016	2.79
Phyllanthin	2.5939	1.38	0.0016	2.79
Guaiazulene	2.8954	1.53	0.0016	2.79
Istamycin AO	8.3592	3.06	0.0016	2.79
7-Hydroxy-6-methyl-8-ribityl lumazine	19.334	4.27	0.0016	2.79
N6-delta2-Isopentenyl-adenine	7.7177	2.95	0.0017	2.78

6-Acetylpicropolin	2.6915	1.43	0.0017	2.77
N-Methylanthranilamide	2.3239	1.22	0.0019	2.73
p-hydroxybenzoate	3.0542	1.61	0.0019	2.71
S-Methyl-3-phospho-1-thio-D-glycerate	5.0161	2.33	0.0019	2.71
Glycoperine	6.1411	2.62	0.0019	2.71
Glycerol 1-phosphate	7.8375	2.97	0.0019	2.71
Pyruvate kinase phosphate	18.306	4.19	0.0019	2.71
Phenylsulfate	4.5312	2.18	0.002	2.71
2S-Amino-tridecanoic acid	3.3953	1.76	0.0021	2.69
2-4-Chlorophenyl-3-phenyl-3-2-pyridinylacrylonitrile	19.24	4.27	0.0021	2.67
S-Substituted N-acetyl-L-cysteine	2.989	1.58	0.0022	2.65
Methazolamide	8.0656	3.01	0.0024	2.62
Estrone glucuronide	3.1205	1.64	0.0025	2.61
BMS-268770	13.48	3.75	0.0025	2.61
1-4-alpha-D-Glucooligosaccharide	6.6574	2.74	0.0026	2.59
6--Dehydro-6--oxoparomamine	8.3311	3.06	0.0026	2.58
3-Oxo-3-ureidopropanoate	8.2451	3.04	0.0027	2.57
Gentisyl alcohol	2.5422	1.35	0.0027	2.56
2-4-Dihydroxypteridine	3.3871	1.76	0.0028	2.56
2-5-Dichloro-4-oxohex-2-enedioate	4.6216	2.21	0.003	2.52
Dinitrogen reductase	3.7192	1.90	0.003	2.52
Perazine	7.6123	2.93	0.003	2.52
S-4-Methylthiobutylthiohydroximoyl-L-cysteine	2.9828	1.58	0.0031	2.51
Uric acid	3.3443	1.74	0.0031	2.51
2-Hydroxy-5-methyl-cis-cis-muconic semialdehyde	7.5825	2.92	0.0031	2.51
N1-Acetylspermidine	73.104	6.19	0.0031	2.51
Apoatropine	2.7146	1.44	0.0032	2.50
Anacyclin	3.5391	1.82	0.0033	2.48
Thiobinupharidine	3.253	1.70	0.0034	2.47
Deisopropylhydroxyatrazine	3.7521	1.91	0.0034	2.47
Metipranolol hydrochloride	2.6621	1.41	0.0034	2.46
Strigolactone ABC-rings	2.9212	1.55	0.0035	2.46
1-1-2-Triphenylpropane	50.089	5.65	0.0035	2.46
Purine	3.3104	1.73	0.0036	2.45
N-Acetylphenylethylamine	3.4987	1.81	0.0036	2.45
WIN IS	13.135	3.72	0.0036	2.45
Bonafousine	2.1797	1.12	0.0036	2.45
Columbin	21.57	4.43	0.0036	2.45
L-2-3-Diaminopropanoate	4.5345	2.18	0.0036	2.44
Crinamidine	5.9837	2.58	0.0036	2.44
Benzimidazole	4.1744	2.06	0.0037	2.43
Kadsurin A	9.5522	3.26	0.0037	2.43
Pimelea factor P2	2.3144	1.21	0.0037	2.43
Bruceine D	2.8774	1.52	0.0040	2.40
Acanthicifoline	10.802	3.43	0.0041	2.39
N-Cyclopropylammeline	18.067	4.18	0.0041	2.38
Eudistomin C	45.913	5.52	0.0041	2.38
Estradiol-17alpha	10.121	3.34	0.0043	2.37
Flutamide	5.8007	2.54	0.0044	2.35
3-Acetyloxy-9-mercaptoandrosta-3-5-diene-11-17-dione	6.8368	2.77	0.0045	2.34

Avizafone	37.212	5.22	0.0045	2.34
Zanamivir	2.8926	1.53	0.0048	2.32
Clozapine	4.2078	2.07	0.0048	2.32
Trimethobenzamide	24.798	4.63	0.0048	2.32
Angustifoline	2.9005	1.54	0.0049	2.31
Metsulfuron methyl	5.8017	2.54	0.0050	2.30
D-glucarate	4.8758	2.29	0.0050	2.30
D-Xylosylprotein	2.1706	1.12	0.0051	2.29
N-Acetylmuramate	2.4655	1.30	0.0051	2.29
1-Ethyl-2-benzimidazolinone	7.9318	2.99	0.0051	2.29
Furofoline I	18.228	4.19	0.0051	2.29
trans_trans-farnesyl diphosphate	6.217	2.64	0.0051	2.29
Pentahomomethionine	23.302	4.54	0.0052	2.29
LL-2-6-Diaminoheptanedioate	18.046	4.17	0.0053	2.28
Nepetalactone trans-cis-form	3.0807	1.62	0.0053	2.28
Acronycidine	1603	10.65	0.0053	2.28
Abyssinone I	6.5505	2.71	0.0053	2.28
Butanoylphosphate	9.5172	3.25	0.0055	2.26
Valproic acid	7.371	2.88	0.0055	2.26
N3--Acetylkanamycin	12.411	3.63	0.0055	2.26
Vulgaxanthin-II	8.3083	3.05	0.0055	2.26
alpha-Amylcinnamaldehyde	4.0836	2.03	0.0058	2.23
Proacacipetalin	9.7704	3.29	0.0061	2.21
2-Hydroxy-6-oxo-6-2-hydroxyphenyl-hexa-2-4-dienoate	1025	10	0.0061	2.21
Amiloride	2.2527	1.17	0.0067	2.17
Procollagen trans-4-hydroxy-L-proline	3.4682	1.79	0.0067	2.17
2-Butylbenzofuran-3-yl4-hydroxyphenylketone	18.075	4.18	0.0067	2.17
Deoxyanisatin	2.7669	1.47	0.0067	2.17
1-2-Anthracenediol	11.922	3.58	0.0068	2.17
Valacyclovir	27.45	4.78	0.0068	2.17
cis-1-2-Dihydro-3-ethylcatechol	32.201	5.01	0.0068	2.17
gamma-Fagarine	28.431	4.83	0.0069	2.16
4-Hydroxymethylphenylhydrazine	13.63	3.77	0.0074	2.13
Quinidine	2.6223	1.39	0.0074	2.13
3-Chloro-L-alanine	3.6925	1.88	0.0074	2.13
Mechlorethamine	5.9008	2.56	0.0074	2.13
2-Polyprenyl-6-hydroxyphenol	19.257	4.27	0.0074	2.13
Amylopectin	8.14	3.03	0.0077	2.11
N4-Acetyl-beta-D-glucosaminylasparagine	8.9674	3.16	0.0077	2.11
2-Amino-4-oxo-6-1--2--3--trihydroxypropyl-diquinoid-7-8-	2.7616	1.47	0.0079	2.10
Norcapillene	3.0381	1.60	0.0079	2.10
4-Oxocyclohexanecarboxylate	6.7593	2.76	0.0079	2.10
L-Octanoylcarnitine	5.6677	2.50	0.0080	2.10
Pseudotropine	8.3221	3.06	0.0082	2.09
Capsaicin	7.0086	2.81	0.0083	2.08
Methoxybrassinin	9.3719	3.23	0.0087	2.06
N-Caffeoylputrescine	0.187	-2.42	0.0087	2.06
Kainic acid	2.0132	1.01	0.0087	2.06
Diethyl phenyl phosphate	2.7216	1.44	0.0087	2.06
Toxoflavine	3.5184	1.81	0.0087	2.06

beta-L-Arabinose 1-phosphate	289.97	8.18	0.0087	2.06
Alcophosphamide	435.98	8.77	0.0088	2.06
